# Tibial tuberosity‐trochlea groove distance is dependent on flexion angle and intra‐articular version in different magnetic resonance imaging recording techniques

**DOI:** 10.1002/jeo2.70300

**Published:** 2025-06-05

**Authors:** Ole Grunenberg, Lucas K. Palma Kries, Michael J. Raschke, Christian Peez, Thorben Briese, Luise M. Hägerich, Lara Leibrandt, Elmar Herbst, Christoph Kittl, Adrian Deichsel

**Affiliations:** ^1^ Department of Trauma, Hand and Reconstructive Surgery University of Muenster Muenster Germany

**Keywords:** instability, MRI, patella, TT‐PCL, TT‐TG

## Abstract

**Purpose:**

The purpose of this study was to compare the tibial tuberosity‐trochlea groove distance (TT‐TG) and the tibial tuberosity‐posterior cruciate distance (TT‐PCL) between conventional and rotatory magnetic resonance imaging (MRI). It was hypothesised that the TT‐TG varies between the investigated techniques, due to differences in knee flexion angle and intra‐articular version. Variations in TT‐TG could lead to misdiagnoses and consequently result in inappropriate surgical indications.

**Methods:**

Twenty‐five patients with both a conventional knee MRI and a rotatory MRI (which allows full knee extension) due to recurrent patellar dislocation were included. TT‐TG and TT‐PCL, knee flexion angle and intra‐articular version (external rotation) were determined in each scan. Inter‐rater reliability was assessed using the intraclass correlation coefficient (ICC). Between‐group differences were assessed using the Wilcoxon matched‐pairs signed‐rank test. Spearman's correlation coefficient was used to detect correlations between the TT‐TG and TT‐PCL with flexion angle and intra‐articular version.

**Results:**

The TT‐TG was significantly (*p* < 0.05) higher in rotatory MRI (median: 18.4 mm, interquartile range [IQR]: 7.3 mm), in comparison to conventional MRI (median: 12 mm, IQR: 5.7 mm), while no significant differences were observed for TT‐PCL. Knee flexion angle was significantly higher in the conventional MRI (median: 21.4°, IQR: 8.6°) compared to rotatory MRI (median: 3.1°, IQR: 3.4°, *p* < 0.0001). The intra‐articular version was significantly lower in the conventional MRI (median: 2°, IQR: 6.3°) compared to the rotatory MRI (median: 9°, IQR: 7.3°, *p* < 0.0001). Measurements showed excellent interrater agreement (ICC: 0.87−0.94).

**Conclusion:**

TT‐TG measurements are dependent on flexion angle and intra‐articular version, which vary with differing MRI techniques. Extension of the knee with a simultaneous higher intra‐articular version in the rotatory MRI technique, suggests increased TT‐TG close to extension, due to the screw‐home mechanism. This should be considered to avoid misdiagnosis due to the TT‐TG.

**Level of Evidence:**

Level III.

**Abbreviations**
CT: computed tomography
ICC: intraclass correlation coefficient
MRI: magnetic resonance imaging
TT‐PCL: tibial tuberosity‐posterior cruciate ligament distance
TT‐TG: tibial tuberositiy‐trochlea groove distance

## INTRODUCTION

Numerous risk factors for recurrent patellar dislocation (RPD) are described in the literature, of which the tibial tuberosity‐trochlea groove distance (TT‐TG) is one of the most frequently used [2, 7, 10, 11, 20, 33, 38]. The TT‐TG was first described by Goutalier et al. in 1978, and was initially determined on X‐rays, but later also on computed tomography (CT) scans [8, 13]. With the better availability of magnetic resonance images (MRI), the TT‐TG was also evaluated for this imaging technique and is now standard in the examination of patellar instabilities [18, 27]. The advantage of utilising MRI, instead of X‐rays, or CT is that it does not involve radiation. However, the use of the TT‐TG in MRI is controversial as the results differ between the two measurement methods and the cut‐off values are not adapted for MRI [3, 16, 32]. Originally, a TT‐TG greater than 20 mm was considered pathological [8]. However, previous studies demonstrated that the original cut‐off value of 20 mm has limited applicability for MRI, leading to the investigation of additional factors, or defining different values as pathological [12, 17, 35].

A possible explanation for varying results between different studies might be heterogeneous recording conditions of the MRI, which are often inadequately described [17, 21, 39]. For MRI of the knee, the knee has to be positioned in an MRI coil, which typically leads to a slightly flexed knee, and free rotation [1]. However, it was shown that the TT‐TG can be influenced by knee flexion [29, 31]. Alternatively, knees can be positioned in a lower extremity coil, with the knee in full extension, similar to CT scans [34]. This technique is commonly used for assessment of rotational alignment of the lower extremities (rotatory MRI) [9]. An alternative to the TT‐TG is the tibial tuberosity‐posterior cruciate ligament distance (TT‐PCL), which was developed to address the problems associated with the TT‐TG [28].

The purpose of this study was to compare two different recording techniques (knee MRI in a knee coil in slight flexion vs. lower extremity coil in full extension) for knee MRI regarding their differences in TT‐TG, and TT‐PCL as well as their correlation with intra‐articular version and knee flexion angle. In addition, the number of patients who falsely exceed the threshold on rotatory MRI should be determined.

It was hypothesised that there is a correlation between the different recording techniques and the TT‐TG, but not between the recording techniques and the TT‐PCL, due to differences in knee flexion between the techniques.

## MATERIALS AND METHODS

This retrospective study was approved by the local ethics committee Ethikkommission der Ärztekammer Westfalen‐Lippe und der Westfälischen Wilhelms‐Universität (File number: 2023‐342‐f‐S). From 2019 to May of 2023, patients with RPD, which received both a conventional MRI and a rotatory MRI, were enroled to this study. Patients were excluded if surgery was performed, or a fracture occurred between the two MRI scans. Furthermore, patients were excluded if more than one year passed between the two MRI scans.

### MRI techniques

The positioning of the knee for the conventional MRI and the rotatory MRI differs considerably between the different MRI techniques as well as between different institutions. The conventional MRI positioning protocol does not require fixed flexion angles of the knee. The conventional protocol stipulates that both rotation and flexion of the knee of up to 20° are permitted and the patient is in supine position, so that comfortable positioning can be achieved. Furthermore, to minimise motion, the knee gets immobilised, using sandbags and straps. However, an exact determination of flexion and rotation is not possible with this positioning. A knee coil is then placed around the examined knee [25].

In contrast, the rotatory MRI protocol includes both legs from the hips to the feet. This helps with the assessment of rotational alignment and is therefore referred to in this paper as “rotatory MRI.” A lower extremity coil is placed around the lower extremities extending from the anterior iliac spine to the feet, which enables, besides the full leg recording, an exact determination of flexion and rotation. Due to the coil, the slices chosen for the rotatory MRI are thicker than in the conventional MRI. The protocol stipulates that the patient is in supine position and in full knee extension, and the ankles are fixed in a neutral position [9, 19]. Rotatory MRIs were only included if both lower extremities from the hip to the ankles were depicted.

### Determination of TT‐TG and TT‐PCL

**Figure 1:**
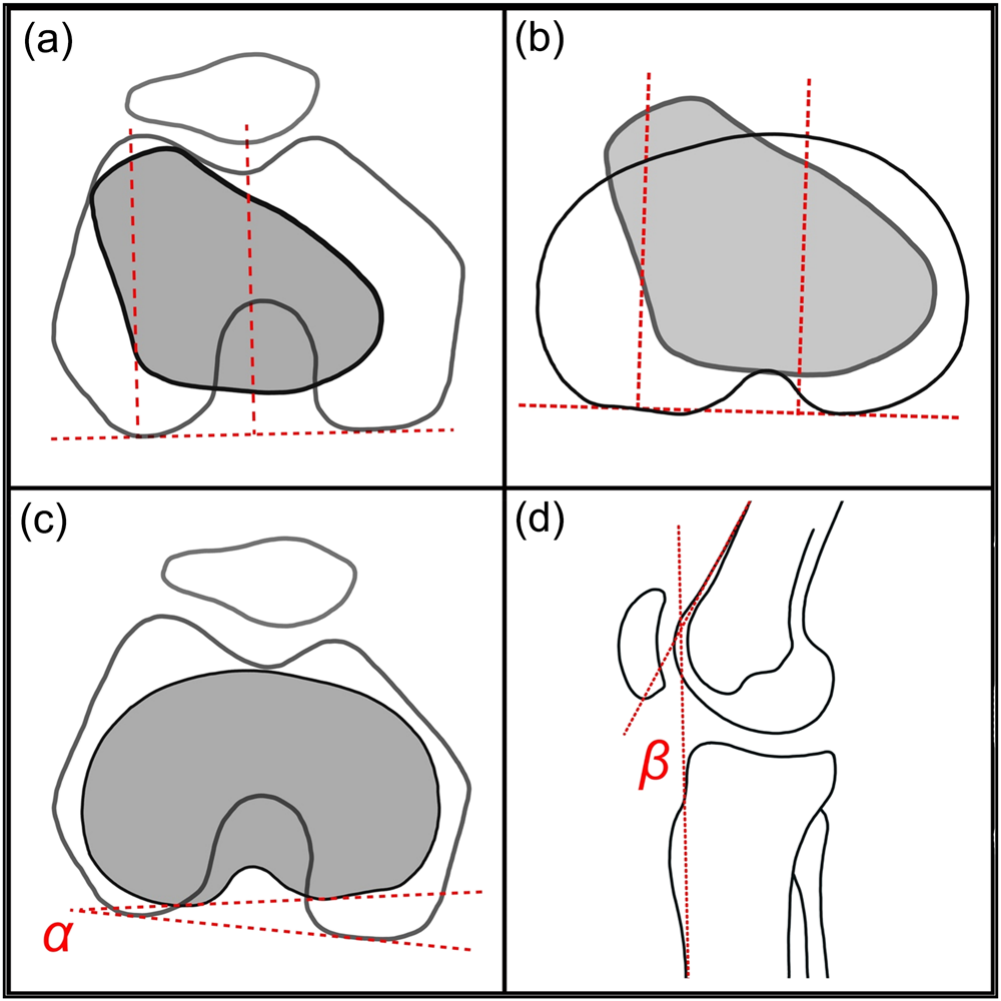
Parameters assessed. (a) Measurement of the tibial tuberosity‐trochlear groove distance. (b) Measurement of the tibial tuberosity‐posterior cruciate ligament distance. (c) Measurement of intra‐articular version (alpha). (d) Measurement of knee flexion (beta).

The MRI‐scans were each analysed by two independent investigators (O.G. and A.D.). The measurements of the TT‐TG and TT‐PCL were carried out using the software Centricity Universal Viewer (GE Healthcare). The measurement methods did not differ between conventional and rotatory MRI scans. The TT‐TG was measured, as previously described [27]. First, a line was placed along the posterior femoral condyles, followed by an orthogonal line that intersected the trochlea groove at its deepest point. This orthogonal line was then transferred to the axial section of the most anterior point of the tibial tuberosity. A parallel line was drawn to this line, intersecting the midpoint of the tibial tuberosity. The distance between these two lines was measured, corresponding to the TT‐TG (Figure 1a).

Analogous, during measurement of the TT‐PCL, the orthogonal line runs along the medial border of the PCL instead of through the trochlear groove. The medial border of the PCL was determined in the most inferior slice it could still be identified. Afterward, the orthogonal line was then transferred to the axial section of the most anterior point of the tibial tuberosity and a parallel line was drawn to this line, intersecting the midpoint of the tibial tuberosity (Figure 1b). The distance between these two lines was measured, corresponding to the TT‐PCL [28].

Since a TT‐TG greater than 20 mm is considered pathological [8], the measurements of the TT‐TG in conventional and in rotatory MRI were compared to this threshold. Furthermore, patients exceeding this threshold in one, but not in the other MRI were analysed.

### Intra‐articular version and knee flexion

The intra‐articular rotation, also known as intra‐articular version, as well as the flexion angle of the knee were measured. Analogous to previous publications, the intra‐articular version was measured in axial MRI. The intra‐articular version was measured as an angle between a tangent at the dorsal tibial head as well as a line through the most posterior point of the medial and lateral femoral condyles (Figure 1c) [19].

The knee flexion angle was determined in the sagittal layer by measuring the angle between a line along the anterior edge of the femur and the anterior tibial crest (Figure 1d), as previously described [30].

### Statistical analysis

All data was collected in Excel (Microsoft). Statistical analysis was performed using Prism (version 10.2.3, GraphPad Software).

Descriptive statistics were performed, and data distribution was tested using the Shapiro–Wilk test, as well as histograms. As data found not to be normally distributed, descriptive data is presented in terms of median and interquartile range (IQR). The two‐tailed Wilcoxon matched‐pairs signed‐rank test was used to compare the assessed parameters between rotatory and conventional MRI. Correlation of TT‐TG and TT‐PCL with intra‐articular version, and flexion angle as well as correlation of intra‐articular version with flexion angle was performed using Spearman's correlation coefficient. A *p*‐value less than 0.05 was deemed to identify significant correlations.

The inter‐rater reliability of the measurements was assessed by using the intraclass correlation coefficient (ICC) in Excel. The results were evaluated according to the guidelines of Ciccetti, with 0.6–0.74 corresponding to a good and 0.75–1 corresponding to an excellent reliability [4].

An a‐priori power analysis was performed using G*Power (version 3.1.9.7, University Düsseldorf). Based on means and standard deviations from a prior study [6], it was assumed that a sample size of 10 would allow the identification of differences in TT‐TG between the two MRI techniques with a mean difference of 4 mm, and a SD of 4 mm (effect size 1), with 80% power, at the significance level of *p* < 0.05.

## RESULTS

Twenty‐five subjects (11 male and 14 female) were included to this study. The mean age of the included patients on the day the conventional MRIs were performed was 20.2 ± 5.5 years and, on the day the rotatory MRIs were performed was 20.3 ± 5.5 years. The mean time between MRIs was 79.4 ± 95 days. The indication for all MRIs was RPD.

**Figure 2:**
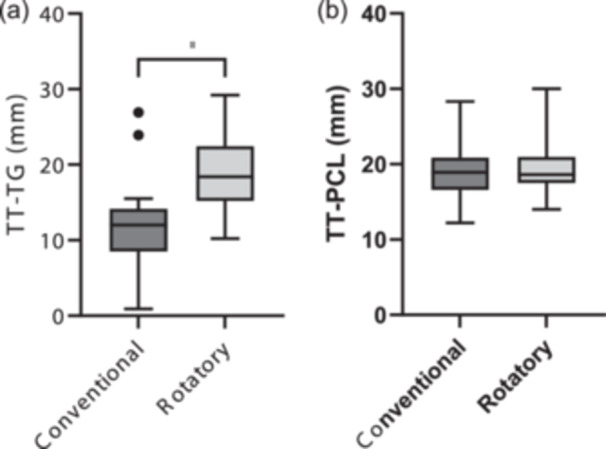
(a) Comparison of the tibial tuberosity‐trochlea groove distance (TT‐TG). (b) Comparison of the tibial tuberosity‐posterior cruciate ligament distance (TT‐PCL) in millimetres between conventional and rotatory magnetic resonance imaging; ****p < 0.0001.

The TT‐TG was found to be significantly lower in the conventional MRIs (median: 12 mm, IQR: 5.7 mm), in comparison to the rotatory MRIs (median: 18.4 mm, IQR: 7.3 mm, *p* < 0.0001, Figure 2a). Seven of the 25 (28%) included patients exceeded the TT‐TG threshold in rotatory MRI, but not in the conventional MRI. No significant difference between the TT‐PCL in conventional MRIs (median: 18.9 mm, IQR: 4.3 mm), in comparison to the rotatory MRIs (median: 18.6 mm, IQR: 3.5 mm, *p* = n.s., Figure 2b) was observed.

**Figure 3:**
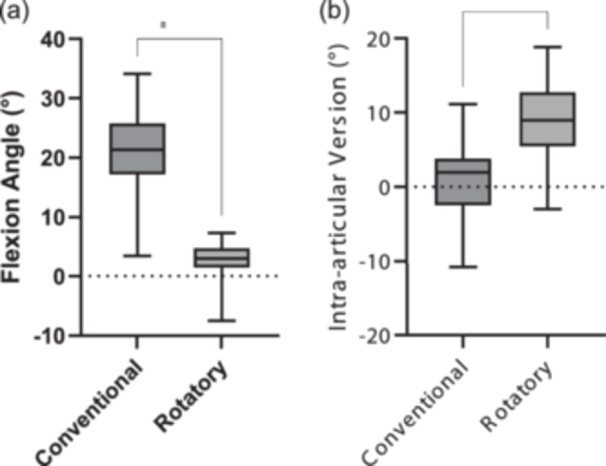
Comparison of the flexion angle (a) and the intra‐articular version (b) in ° between conventional and rotatory magnetic resonance imaging; ****p < 0.0001. Positive values of the intra‐articular version correspond to external rotation.

The knee flexion angle was found to be significantly higher in conventional MRIs (median: 21.4°, IQR: 8.6°), in comparison to the rotatory MRIs (median: 3.1°, IQR: 3.4°, *p* < 0.0001, Figure 3a). The intra‐articular version was significantly lower in the conventional MRI (median: 2°, IQR: 6.3°) compared to the rotatory MRI (median: 9°, IQR: 7.3°, *p* < 0.0001, Figure 3b).

**Figure 4:**
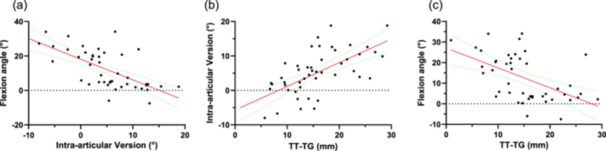
Correlation of the knee flexion angle, the intra‐articular version, and the tibial tuberosity‐trochlea groove distance (TT‐TG). (a) Correlation between knee flexion in ° and intra‐articular version in °. (b) Correlation between intra‐articular version in ° and the TT‐TG in millimetres. (c) Correlation between knee flexion in ° and the TT‐TG in millimetres.

A statistically significant negative correlation was found between the intra‐articular version and the knee flexion angle (*r* = −0.69, 95% confidence interval [CI] = −0.82 to −0.49, *p* < 0.0001, Figure 4a), a significant positive correlation was found between the TT‐TG and the intra‐articular version (*r* = 0.67, 95% CI = 0.46–0.8, *p* < 0.0001, Figure 4b) and a significant negative correlation was found between the TT‐TG and the knee flexion angle (*r* = −0.56, 95% CI = −0.74 to −0.31, *p* < 0.0001, Figure 4c).

There were no significant correlations between the TT‐PCL and the knee flexion angle (*r* = 0.09, 95% CI = −0.22 to 0.38, *p* = n.s.), the TT‐PCL and the intra‐articular version (*r* = 0.003, 95% CI = −0.29 to 0.29, *p* = n.s.) and the TT‐PCL and TT‐TG (*r* = 0.25, 95% CI = −0.05 to 0.5, *p* = n.s.).

All measurement parameters of the TT‐TG in conventional MRIs (ICC: 0.87) as well as in rotatory MRIs (ICC: 0.91), and of the TT‐PCL in conventional MRIs (ICC: 0.94) as well as in rotatory MRIs (ICC: 0.92) showed excellent inter‐rater agreements.

## DISCUSSION

The most important finding of the present study was that different MRI techniques in the same knee led to markedly different TT‐TG measurements, while the TT‐PCL was not significantly influenced. Thus, the hypothesis can be accepted. Furthermore, a decreased flexion angle was accompanied with an increased intra‐articular version and thus with an increased TT‐TG which could be observed in rotatory MRI scans.

Historically, the TT‐TG was developed by using X‐rays by Goutalier et al. [13]. In the following, the TT‐TG was also used in CT scans and a pathological threshold of 20 mm was introduced by Dejour et al. to identify patients with RPD [8]. This threshold was determined in full knee extension. However, the threshold of 20 mm prevails amongst clinical studies (including studies utilising MRI), and is frequently used as an indication for tibial tubercle osteotomy [8, 23, 24].

Due to the increasing use of MRI and the predominantly young patients with patella dislocation, the TT‐TG was more frequently used in MRI scans. However, measuring the TT‐TG in MRI is controversial [3, 16, 32, 37]. Above all, the use of the same threshold as for CT images has been criticised in previous studies [32].

A previous clinical study investigating 21 patients, measured the TT‐TG in sequential MRI. It was found that a significant correlation between the TT‐TG and the tibiofemoral rotation in paediatric patients exists which is in line with the results of our study [26]. However, the previous work could not provide explanations for this phenomenon and concluded that the values differ because of the changes of the tibiofemoral rotation in paediatric patients. The current study found that these differences also occur in adults and that the intra‐articular version changes is correlated with the flexion angle. As this previous paper did not mention the positioning of the knee, it cannot be ruled out that the flexion angle changed in between sequential recordings.

Further studies have shown that knee flexion can alter the TT‐TG. A study with eight patients examined the effects of the knee flexion angle and weight‐bearing on the TT‐TG. It was concluded that the TT‐TG decreases by increasing knee flexion as well as during weight‐bearing in 0° but not in 30° knee flexion [21]. Another study measured the TT‐TG in 26 knees of adolescents in 30°, 20°, 10° and 0° of knee flexion. The decrease of the TT‐TG by increasing flexion is in agreement with the previous and with the current study [31]. However, both studies did not analyse the intra‐articular version in different flexion angles so that the reason for these effects can only be speculated. Based on the present study, which showed that both intra‐articular version and TT‐TG correlated significantly with knee flexion, it can be concluded that an increased TT‐TG in knee extension is caused by the screw‐home mechanism of the knee, which occurs at the last 20°–30° of knee extension. During the screw‐home mechanism, the greater articular surface of the medial condyle of the femur compared to the lateral condyle leads to prolonged anterior gliding of the medial tibia [14, 22]. Therefore, the tibia rotates externally as well as the tibial tubercle, which becomes apparent by an increasing TT‐TG as the trochlea groove keeps its position.

The present study is of relevance due to its implications for clinical practice. Most clinical studies, determining the TT‐TG in MRI images, do not report on the type of MRI imaging method used, or the flexion of the knee [17, 21, 39]. This and the notion that the pathological threshold of 20 mm was determined using CT images makes it difficult to assess whether a TT‐TG is truly pathological, or not. The present study indicated that measurements of the TT‐TG exceeded or fall below the threshold due to the degree of flexion alone which is a major weakness of this parameter [31]. Therefore, the clinician should be wary of pathological TT‐TG values and must always consider the way MRI scans were taken, as possible confounders. Seven patients of the present study would exceed the threshold on rotatory MRI, but not on conventional MRI. The selection of the MRI technique alone could thereby create an indication for surgery by erroneously exceeding the threshold. Furthermore, additional risk factors for RPD should be considered, before indicating surgery like tibial tubercle transfer [36]. The problem of distortion of the TT‐TG by varying flexion angles could be addressed by adhering to a standard protocol of how to perform knee MRIs in patients with patellar instability, as previously demanded by other authors as well [31]. Pathological thresholds for patients with patellar instability could then be re‐established in future studies.

Since the TT‐TG was increasingly criticised, Seitlinger et al. developed a new parameter that should assess the lateralisation of the tibial tuberosity independently of the knee flexion [28]. However, subsequent studies also showed weaknesses in this parameter. A following study analysed the TT‐TG and the TT‐PCL and concluded that the TT‐PCL is less predictive of recurrent patella instability [15]. Furthermore, the TT‐PCL was evaluated in a paediatric cohort and showed that this parameter did not correlate with patella instability [5]. Although the TT‐PCL was not susceptible to the same distortions as the TT‐TG in the present study, it is therefore still questionable whether this parameter should be preferred to the TT‐TG, due to its limited clinical correlation. However, the TT‐PCL can be determined as an adjunct parameter, additionally to TT‐TG, and several others [15]. In the present study, both TT‐TG and TT‐PCL, showed excellent inter‐rater reliability, which is in accordance with previous studies [10, 15].

This study suffers from several limitations that need to be considered. As it had already been criticised in previous studies, no uniform protocol was carried out for the conventional MRI scans, as MRI scans were performed by different radiologists. This could effect the thickness of the slices as well as positioning of the knee. Furthermore, all included subjects presented with RPD, as only these patients had both a conventional and rotatory MRI scans performed. Lastly, without a healthy control group, no conclusions about pathological thresholds can therefore be drawn from this study.

## CONCLUSION

TT‐TG measurements are dependent on flexion angle in intra‐articular version, which vary with differing MRI techniques, with clinically relevant differences. The TT‐PCL is not influenced by these factors. Extension of the knee with a simultaneous higher intra‐articular version in the rotatory MRI technique, suggests increased TT‐TG close to extension, due to the screw‐home mechanism.

## AUTHOR CONTRIBUTIONS

All listed authors contributed substantially to this work (Ole Grunenberg for the acquisition of data, MRI analysis, statistical analysis, and writing. Adrian Deichsel for the acquisition of data, idea and study design, MRI analysis, statistical analysis, and writing. Lucas K. Palma Kries for the MRI analysis. Lara Leibrandt for the preparation of figures. Christian Peez, Thorben Briese, Luise M. Hägerich, Michael J. Raschke, Christoph Kittl, and Elmar Herbst for the internal review) and approved the submission to KSSTA.

## CONFLICT OF INTEREST STATEMENT

Elmar Herbst is Deputy Editor‐in‐Chief for the Knee Surgery, Sports Traumatology and Arthroscopy (KSSTA). Adrian Deichsel is Web Editor for the Knee Surgery, Sports Traumatology and Arthroscopy (KSSTA). The remaining authors declare no conflicts of interest.

## ETHICS STATEMENT

The specimens were dissected and biomechanically tested under the approval of the “Ethikkommission der Ärztekammer Westfalen‐Lippe und der Westfälischen Wilhelms‐Universität” (File number: 2023‐342‐f‐S).

## Data Availability

Data are available from the corresponding author upon reasonable request.
